# Patient perspectives on buprenorphine/naloxone for chronic noncancer pain: A qualitative interview study

**DOI:** 10.1080/24740527.2026.2627924

**Published:** 2026-03-26

**Authors:** Katelyn Halpape, Derek Jorgenson, Marla Rogers

**Affiliations:** a College of Pharmacy and Nutrition, University of Saskatchewan, Saskatoon, Canada; b Canadian Hub for Applied and Social Research (CHASR), University of Saskatchewan, Saskatoon, Canada

**Keywords:** Buprenorphine/naloxone, low-dose initiation, chronic pain, patient perspective, qualitative research

## Abstract

**Background:**

Buprenorphine/naloxone (Suboxone) is an emerging option for chronic noncancer pain (CNCP), but evidence on transitioning patients from full opioid agonists remains limited. At the University of Saskatchewan Chronic Pain Clinic (UCPC) in Saskatchewan, Canada, pharmacists support these transitions within a unique interdisciplinary model.

**Aim:**

The aim of this study was to explore patient perspectives on transitioning to and using buprenorphine/naloxone for CNCP.

**Methods:**

This qualitative study involved one-on-one semistructured interviews with current and former UCPC patients who had transitioned to buprenorphine/naloxone for CNCP. Participants were invited by email to complete semistructured interviews via Zoom or telephone with a trained facilitator. The interview guide was informed by a literature review and pretested. Recorded interviews were transcribed using an artificial intelligence–supported platform, reviewed for accuracy, and analyzed in NVivo 14 using thematic analysis.

**Results:**

Seven participants (five females, two males; average age 57 years) were interviewed. Most had used opioids long term for pain prior to transition. Experiences varied: some transitioned easily, whereas others required more time or experienced discomfort. Buprenorphine/naloxone tablet size and taste were the most common negative experiences. Side effects were mild (e.g. drowsiness, constipation). Some expressed concern about long-term use and desired clearer communication during transitions. Although buprenorphine/naloxone was not uniformly experienced as effective for pain relief, most would recommend buprenorphine/naloxone to others.

**Conclusion:**

Patient experiences at UCPC suggest that though buprenorphine/naloxone is not a universal solution for CNCP, it may be an effective option for carefully selected individuals. Side effects were tolerable, and most would recommend the treatment. Improved communication during transitions may enhance patient experiences.

## RÉSUMÉ

**Contexte :** La buprénorphine/naloxone (Suboxone) constitue une option émergente pour le traitement de la douleur chronique non cancéreuse (DCNC), mais les données probantes sur la transition des patients traités par des agonistes opioïdes complets vers ce traitement demeurent limitées. À la Clinique de la douleur chronique de l’Université de la Saskatchewan (UCPC), en Saskatchewan (Canada), les pharmaciens soutiennent ces transitions dans le cadre d’un modèle interdisciplinaire unique.

**Objectif :** Le but de cette étude était d’explorer les points de vue des patients sur la transition vers la buprénorphine/naloxone et sur l’utilisation de ce traitement pour la douleur chronique non cancéreuse.

**Méthodes :** Cette étude qualitative a consisté en des entretiens individuels semi-structurés avec des patients actuels et anciens de l’UCPC ayant fait la transition vers la buprénorphine/naloxone pour la douleur chronique non cancéreuse. Les participants ont été invités par courriel à participer à des entretiens semi-structurés par Zoom ou par téléphone avec un animateur qualifié. Le guide d’entretien s’est appuyé sur une revue de la littérature et a été prétesté. Les entretiens enregistrés ont été transcrits à l’aide d’une plateforme assistée par intelligence artificielle, puis vérifiés pour en assurer l’exactitude et analysés dans NVivo 14 au moyen d’une analyse thématique.

**Résultats :** Sept participants (cinq femmes et deux hommes; âge moyen : 57 ans) ont été interrogés. La plupart des participants avaient utilisé des opioïdes à long terme pour soulager la douleur avant la transition. Les expériences étaient variées : certains participants ont effectué la transition facilement, tandis que d’autres ont eu besoin de plus de temps ou ont ressenti de l’inconfort. La taille et le goût des comprimés de buprénorphine/naloxone constituaient les expériences négatives les plus fréquemment mentionnées. Les effets secondaires étaient légers (p. ex. somnolence, constipation). Certains participants ont exprimé des préoccupations quant à l’utilisation à long terme et ont souhaité une communication plus claire pendant la transition. Bien que la buprénorphine/naloxone n’ait pas été perçue comme uniformément efficace pour soulager la douleur, la plupart des participants recommanderaient ce traitement.

**Conclusion :** L’expérience des patients à l’UCPC indique que, bien que la buprénorphine/naloxone ne soit pas une solution universelle pour la douleur chronique non cancéreuse, ce traitement peut constituer une option efficace pour des patients soigneusement sélectionnés. Les effets secondaires étaient tolérables, et la plupart des participants recommanderaient ce traitement. Une meilleure communication pendant les transitions pourrait améliorer l’expérience des patients.

## Introduction

Chronic pain affects approximately one in five Canadians and poses a substantial burden on individuals and the health care system.^1,2^ For many individuals, long-term opioid therapy (LTOT) has been a mainstay of treatment despite limited evidence of sustained benefit in chronic noncancer pain (CNCP).^3–5^ Though some patients may derive benefit from LTOT as part of CNCP management, there are also risks that, for many patients, may outweigh these benefits including opioid-induced hyperalgesia, physical dependence, constipation, sedation, endocrine dysfunction, mood disturbances, and increased risk of overdose.^3,5^ As clinicians and health systems seek safer, more effective alternatives for managing CNCP, buprenorphine/naloxone (Suboxone) has emerged as a promising option.^6–8^

Buprenorphine is a partial μ-opioid receptor agonist that is formulated with naloxone, an opioid antagonist to minimize diversion, which has traditionally been used in the treatment of opioid use disorder (OUD).^6,7,9^ More recently, it has gained traction as a potential analgesic option for individuals living with CNCP.^5–7,9^ Buprenorphine offers a unique pharmacologic profile that may reduce the risk of respiratory depression, overdose, and opioid-related adverse effects while providing analgesia.^5–7,9^ However, its analgesic effects are variable, with some studies demonstrating meaningful pain reduction when switching from LTOT and others showing little to no difference in pain outcomes compared with remaining on LTOT.^8,10,11^ Furthermore, much of the motivation behind switching to buprenorphine/naloxone from LTOT stems not only from clinical considerations but also from social concerns surrounding opioid use and the ongoing opioid crisis.^12^ Thus, evidence to guide transitions from full opioid agonists to buprenorphine/naloxone for CNCP remains limited, because most information relates to its use for OUD.^13^ Specifically, though low-dose or microdosing initiation strategies are increasingly used to ease transitions and avoid precipitated withdrawal, little is known about how patients experience these transitions or how they perceive buprenorphine/naloxone for pain relief in real-world settings.^13^

To date, only one qualitative study has explored this experience in detail, focusing on a veteran population in the United States.^14^ In Canada, this knowledge gap remains largely unaddressed. The University of Saskatchewan Chronic Pain Clinic (UCPC) offers a unique interdisciplinary model of care, operated primarily by pharmacists with limited but strategic involvement from remote pain specialist physicians.^15^ This team-based approach leverages the expertise of pharmacists, physical therapists, and social workers to support patients living with CNCP across Saskatchewan.

A distinctive feature of the UCPC model is that it does not assume prescribing responsibility. Instead, the UCPC pharmacist and specialist physician collaborate to develop individualized care plans that are shared with the referring primary care provider (i.e., family physicians and nurse practitioners), who then writes the prescriptions required to implement care plans. These plans include detailed prescribing guidance and are accompanied by education, resources, and ongoing mentorship to support primary care providers in initiating and sustaining recommended treatments. Most services at UCPC are delivered virtually. The UCPC team meets with patients to assess their CNCP, discuss treatment goals, provide education, and codevelop care plans. This model has been well received by both clinicians and patients.^16,17^ In a recent health professional survey, the UCPC interdisciplinary model was found to increase provider confidence in delivering CNCP care, including opioid therapy management.^16^

Several UCPC patients have been successfully transitioned from LTOT to buprenorphine/naloxone, but their lived experiences during and after this transition have not been formally studied.^18,19^ This study aimed to qualitatively explore the experiences of patients with CNCP who were transitioned from full opioid agonists to buprenorphine/naloxone within the UCPC interdisciplinary model.

## Materials and methods

### Research questions

This study examined patient experiences transitioning from LTOT to buprenorphine/naloxone within the UCPC model. Specifically, the research examined the challenges and facilitators patients encountered during the transition, as well as their perceptions of how the change affected their pain management, functioning, and overall quality of life. In addition, the study explored the psychological and emotional responses patients reported throughout the transition, their coping mechanisms and self-management strategies, and the supports they relied on. Finally, the research considered patients’ recommendations for improving the transition process.

### Methodology, ontology, and epistemology

Given the paucity of research regarding patient experiences in transitioning from LTOT to buprenorphine/naloxone for CNCP, a qualitative descriptive study was conducted. This orientation was chosen because it prioritizes clear, practice-relevant accounts of participants’ perspectives.^20,21^ Ontological and epistemological commitments were therefore aligned with qualitative description to capture and summarize experiences without imposing interpretive or theoretical frameworks.

It was a priority in this study to give voice to the participants without the influence of clinicians involved in their care. The use of semistructured interviews with broad, open-ended questions allowed participants to describe their experiences in their own terms. The interviewer and data analyst had no clinical training or background in LTOT, buprenorphine/naloxone, or CNCP prior to conducting the interviews, which minimized professional or disciplinary bias during data collection and analysis. Although the interviewer asked follow-up questions to clarify details of participants’ experiences, each interview concluded with an intentionally open prompt (“Is there anything else about your experience you want us to know that we haven’t already asked you about?”) to elicit concerns or insights not anticipated by the study team. By centering participants as the primary experts on their own transitions, the study aimed to generate foundational, patient-driven insights into an underexplored area of CNCP care.

### Interview guide

The interview guide (Appendix) was developed by the research team based on a review of the literature. In particular, the guide used in a similar study by Edmond et al. informed several questions.^14^ Edmond et al. explored patient experiences when transitioning from full-agonist LTOT to buprenorphine within two Veterans Affairs systems using semistructured interviews.^14^ Their guide included ten core questions and probes addressing chronic pain history, transition processes, stigma, and perceived outcomes.^14^ Permission was obtained to adapt this guide for use in the present study.

### Participants

All UCPC patients who had been transitioned to buprenorphine/naloxone between March 2020 (when UCPC opened) and September 2024 (when recruitment materials were distributed) were eligible to participate. Recruitment emails were sent by the UCPC medical office assistant, inviting interested patients to contact the Canadian Hub for Applied and Social Research (CHASR).

### Interview procedures

An invitation email was distributed to eligible current and former UCPC patients on September 3, 2024, inviting them to participate in an approximately 30-min interview. A reminder email was sent on October 15, 2024. All participants were emailed study information in advance. At the beginning of each interview, the interviewer reviewed the study objectives, answered any questions, and obtained verbal consent.

A CHASR staff member with over 10 years’ experience facilitating semistructured interviews conducted and recorded all interviews using a University of Saskatchewan (USask) Zoom account. One interview was conducted via telephone for a participant who did not have computer access. An honorarium of $50 was provided to each participant immediately after their interview via electronic bank transfer. Recordings were transcribed using an artificial intelligence–supported platform (Rev.com) and reviewed for accuracy by trained CHASR personnel.

Transcripts were provided to participants for review, with two participants requesting minor edits and the remainder approving their transcripts as written. The interviews occurred between October and December 2024.

### Analysis

Transcripts were uploaded to NVivo 14 for analysis.^22^ Data were analyzed using an inductive, exploratory thematic analysis. First, transcripts were read in full without coding to establish familiarity with the data. Second, broad parent codes reflecting the interview guide were created to organize the data set. Third, inductive coding was used to generate codes directly from patient language. These codes were then refined and consolidated into broader themes that captured the patterns across the dataset. The analysis was descriptive and aimed to produce a low-inference account of participant perspectives rather than develop theory. Coding and theme development were conducted by an independent qualitative analyst with prior experience in health-related research but no formal health care training, which may have reduced the likelihood of clinical assumptions shaping interpretation. Field notes were used as supplemental context, with transcripts remaining the primary data source. The study protocol was approved by the University of Saskatchewan Research Ethics Board (Beh 4967).^23^

### Sample size

Seven participants were included in this study. This sample size is consistent with methodological guidance for qualitative research, which aims to obtain rich, detailed accounts of experience rather than statistical generalizability. Prior literature suggests that even relatively small samples can yield sufficient depth to identify common patterns, highlight divergences, and generate meaningful insights, particularly when participants engage in focused, semistructured interviews.^24^ Given the study’s emphasis on depth and descriptive thematic analysis, the sample was considered adequate to address the research aims.

## Results

### Participants

Seven patients participated in the interviews: five women (71.4%) and two men (28.6%). Their ages ranged from 32 to 69 years, with a mean age of 57.4 years. Most (*n* = 6) lived in an urban area. The interview recordings ranged from 22 to 38 min (mean = 28.5 min).

Participants were transitioned to buprenorphine/naloxone between 2021 and 2023, with one in 2021, four in 2022, and two in 2023. All but one participant was still taking buprenorphine/naloxone at the time of the interview; this participant had taken it for just over 1 year and then stopped. Opioid use prior to switching to buprenorphine/naloxone varied: three participants had been prescribed opioids (e.g., hydromorphone, fentanyl, tramadol) for more than 10 years, two participants for 3 to 6 years, and one for less than a year. One participant had not previously used opioids and had been treated with gabapentin, which was switched to pregabalin, prior to transitioning.

### Interview findings

Thematic analysis generated eight overarching themes that captured participants’ experiences of transitioning to buprenorphine/naloxone: (1) motivations for switching; (2) delays to starting and system-level barriers; (3) supports and facilitators to switching; (4), transition experiences and challenges; (5) side effects; (6) pain, quality of life, functioning, and stigma; (7) expectations; and (8) suggestions for improvement (Table 1).

**Table 1.** Interview themes and subthemes with supporting quotes.

Theme	Subtheme	Supporting quotes
Motivations for switching	The choice to switch was driven by internal motivations	*“Well, just being on that other stuff for long just doesn’t make you the best person in the world or whatever, and it just seemed like they weren’t handling the pain. I wasn’t feeling any better.”* *“Since I knew that my recovery was going to be pretty lengthy … I was kind of looking for a more long-term medication that I could be on consistently that would help me through those times instead of just going on and off something constantly or being on something that made me feel worse, I guess.”*
Delays to starting and system-level barriers	Prescribing barriers	*“That’s another thing that makes me laugh here, too, the hydromorphone, anybody can prescribe it, and any pharmacy can fill it, but the Suboxone, you have to go through this big hoop and everything.”*
	Insurance or pharmacy barriers	*“But it’s my own insurance company that caused the most issues. And still to this day, they won’t let her bill a month at a time. And [my pharmacist] said if someone comes in with an addiction, they’ll let her bill a month at a time, but for pain management, they’re not—it’s just, she said she totally doesn’t understand, and this is a new insurance provider for us. So I haven’t had a great experience with them.”*
	Delays were linked to certainty	*“They keep on asking you questions to make sure you want to try it, make sure you want to try it to keep on, just asking questions. And, I don’t know, it took me almost a year.”* *“This sounds kind of cheesy, but for me personally, I wish that the process to actually—when you decide you’re going to do it and when you actually get to do it, not to let that drag on.”*
	Information gaps	*“I kind of feel like I was kind of blindsided a little bit, too. I think they like to get people off of the opiates and that there, and I think they kind of proffer this false hope and it gets people off because they’re willing to try something different and they want to get off. But then in my case, I found out that it was one of the biggest grifts going out there because it didn’t do anything for my pain. It just moved me over from one thing that didn’t work to another thing that didn’t work.”* *“Yeah, the hardness of trying to get off of it. The steps that I found are essentially the same as getting off the hydromorphone.”*
Supports and facilitators to switching	Health professional support	*“I found them really helpful because I didn’t really know a lot about Suboxone to begin with, and I also didn’t really know a lot about the medications that I was on. And so they did a really good job of just explaining what these different medications are and how they can help. And so I think I chatted with someone on the phone a couple times and then had some video appointments, and they were really good with answering my questions.”*
	Reassurance	*“Had the guarantee from my physician that if it didn’t work, I could go back to what I was on. So some of those things just turned out to be security blankets.”*
Transition experiences and challenges	Physical experience of transition	*“Second or third day, I went through a tough day pain-wise, and I can remember thinking that I didn’t think this was going to last. I didn’t think I was going to go through with this, that I was going to stop. But by the next day I was feeling better and virtually went through the rest of it. Didn’t feel any withdrawal or anything like that, just a subtle change and having to work on my mental status through that process.”* *“Let’s say the ninth or tenth day, I had a revelation mode where I realized that something really was changing and that I really was feeling better in ways I just didn’t really recognize to start with.”*
	Initial optimism or hope	*“Excited feeling about it, yeah. I figured it would take at least 6 weeks to really have a good taste of it, to see if it was going to work. I thought that was reasonable, 6 weeks, and I thought it really worked well for me at first, and it’s only the last about 2 months that I feel that I need to increase.”*
	Disappointment or perceived ineffectiveness	*“It’s just that the whole purpose here or whatever, which I think is what patients expect and what the health care providers want to give, is just not there. All the logistics were there. I mean, it’s kind of like hiring a crew. You paint this house and you put new floors in, and concrete driveway—it’s beautiful like that. And then the next day you look and the house is falling apart because the bones were broken. And that’s the whole thing, like the logistics and getting everything, talking about this and that, and the process, and getting the doctor ready with it. And then all of this sort of stuff was there, but the bones of the program were just made out of sawdust because it did nothing. So yeah, that was really unfortunate.”* *“Nothing changed, nothing changed. The hydromorphone wasn’t working anymore. My pain was basically [the same], it stopped working. I was taking the medication and my pain was all there. When I switched over to Suboxone, nothing changed.”*
Side effects	Mood, sleep, and psychological effects	*“If it helps with the pain, fine. But like I said, the mental anguish that it put you through to me is not worth it. And that’s their strong selling point is it’s going to help you with the pain, but there is no mention of what kind of negative effects it could be on it.”* *“But if I go off of it, then I get some withdrawal, like very, very bad depression. And it’s more the depression, it’s just overwhelming. So that’s why I stay on it, but it doesn’t really help.”* *“Everybody noticed my personality and stuff changed it. I was very short with people and just nervous, I guess, a lot of times with it.”*
Pain, quality of life, functioning, and stigma	Improved mood, energy, and mental clarity	*“Generally, overall my mood is better.”* *“Sleeping well, my appetite is better. Pain feels better. My mind is a lot clearer, like I don’t feel like I’m on a pain medication, even though I am.”*
	Positive impact on quality of life	*“I actually went through a period of time there where I would tell my family, I’m not high on this drug because I was so giddy and just to show my husband, I would then dance around the living room and do silly things like that and just truly felt like I had a life back that I hadn’t seen for many, many, many years. Never, ever thought I could have that back.”* *“Well, I believe it’s got better because I can talk to people now and I can go out to different activities, and it’s kind of freed me to even—I feel safe enough even going for a drive by myself to meet up with my girlfriend or something.”* *“It’s changed a lot. Mostly because, I mean, there’s obviously because I’m not in pain, but also just mentally I feel like I don’t have to worry about what I’m taking, if that makes sense.”* *“Like I said, I can be more active and more able to do things with my daughter. And my stamina … being able to stand for longer periods of time without having to sit. The nerve pain in my leg isn’t near as intense, so the nerve pain/numbness, it makes it sometimes itchy and I don’t feel as itchy. I’m not scratching myself raw like I used to.”*
	Negative impact on quality of life	*“Well, and I think that’s—again, I feel like my quality of life in some ways has been worse because now I’m dealing with anxiety, but that isn’t because of the Suboxone. That’s because of coming off those other drugs. But it all happened at the same. … But I feel like dealing with anxiety now and that before I was just dealing with pain, I’d much rather deal with pain than anxiety.”* *“I think it went downhill a little bit because of just what it did to me. Kind of brought the bat out on me, shortness and anger and stuff like that.”*
	Physical and functional improvements	*“It’s probably a combination of medication plus all the work that I did with physio and stuff like that, that has improved my abilities and function and stuff like that. But it definitely helps to be on a medication that doesn’t make you feel crappy and doesn’t make you feel sleepy because then I felt like I could actually focus on my physio and actually do the exercises.”* *“I feel like I can do things a little bit more. I have a little bit more tolerance to the pain. The dullness isn’t quite as intense, so I can do more things, more activities that I’ve had to give up previously because of the pain or wasn’t doing because I chose not to hurt myself.”*
	Effective pain management	*“Quite effective.”*
	No pain management	*“Suboxone was one of the—I mean, it was kind of false hope. I had hoped that it would—because it doesn’t do anything for my pain at all. It doesn’t. I’ve tried to come off the Suboxone and then of course I get withdrawal symptoms or whatever, which are horrifying. But in terms of pain control, it doesn’t really do anything.”* *“Not really, no. It’s basically—to be honest with you, getting off the hydromorphone, fentanyl, and Suboxone and everything—I mean, the pain is still there, but I don’t think that it helped a whole bunch with the pain.”*
	Partially effective pain management	*“I’m torn, because I don’t want to suggest that Suboxone’s not a good drug because it made such a difference in my life. But I am having to deal with more pain. I’ve gone back and asked for it to be increased by 2 milligrams.”* *“It worked really well right at the beginning and it’s like it’s—I need an increase of it now. Because it’s not working as well as I think it should.”* *“In my mind, am using the Suboxone to manage the anxiety and Tylenol to manage the pain, but I guess you can’t differentiate what’s doing what for sure. But my pain is better than it was. It’s better managed. But it’s also very interesting, is if my anxiety is bad, my pain is less, if my anxiety is less, my pain is more.”* *“It kind of takes the edge off a very, very sharp pain in my spine and makes it dull instead of sharp, if that helps. If that makes sense. It doesn’t go away, but it’s not as jarring.”*
Expectations	Exceeded expectations	*“It far exceeded my expectations. Never expected to have this amount of decreased pain. I found that the transition was good. I found that the side effects were next to nil, so it far exceeded my expectations.”*
	Met expectations	*“I think it compares pretty well, once I actually knew what it does and how it can help and stuff like that. I kind of had, I don’t know, I would say reasonable expectations going into it, so I think they met the expectations.”* *“I believe that at the beginning I believe that it was going to be the answer for my pain problem. And I also understand that over time you might have to increase medication no matter what it is. And so I just expected that it would have to increase. And it’s been, well, quite a while I’ve been on it now, and so to increase it, it didn’t surprise me that I might have to do that.”*
	Disappointment or mixed views	*“I don’t know that I had big expectations, except I don’t think that I expected that I would be on it long term. And I think the plan was that I was going to be on it short term, but then it became evident that I couldn’t get off of it.”* *“But I guess I had hopes, but just nothing came to fruition.”* *“I wasn’t impressed.”*
Suggestions for improvement	Provide more information or clarity	*“But nobody really ever said, ‘This is Suboxone, this is what it is, this is what it does, this is what you should expect.’ Nobody ever said that. And maybe the reason why is because maybe it doesn’t have a good track record.”*
	Educational tools or forums	*“I wish they would have something like a forum and whether it’s on Zoom, on the computer or whatever, but just so people can be together, maybe a dozen people, and be introduced to it and then hear about the pros and cons and how other medications would affect you compared to this. And then they would have a chance to ask questions.”*
	Long-term planning and follow-up	*“I guess just making sure that it’s clear, are you doing this or long-term? And is there any risks of being on these medications long term? I think that the biggest, and I think [my health care provider] did say ‘Yes, you can stay on it,’ but then I feel like nobody’s following up on that now.”*
	Side effects and tapering	*“A great help to me in making the decision would’ve been to explain some of the bad things or negative feelings that can develop or something like that. And then also to explain to ’em that it is really not much difference in trying to wean yourself off of an opioid like hydromorphone or fentanyl. You still have to fight that battle at the end of your Suboxone run.”*

Suboxone: Buprenorphine/naloxone, N/A: not applicable.

### Theme 1: Motivations for switching to buprenorphine/naloxone

Participants were driven to switch to buprenorphine/naloxone by a mix of internal motivations and external circumstances. For nearly all participants, a UCPC clinician—most often the pharmacist—was the first to introduce buprenorphine/naloxone as an option. One participant first heard about it from a UCPC physical therapist and another from a UCPC social worker.

Several participants described dissatisfaction with how they felt on their existing opioid regimen (*n* = 3). One participant reported feeling “high” and unwell, noting that her previous medication “messed with my stomach … like, I didn’t feel very good when I was on it.” Another described being “very sick” and no longer experiencing any analgesic benefit. Two others also felt their current medications, which where hydromorphone and tramadol, had become ineffective over time.

For one participant, education about the long-term consequences of opioid use shifted her willingness to consider a change to buprenorphine/naloxone. She previously “froze up the minute anyone wanted to talk to me about changing my fentanyl” because it was “the only thing … that had helped at all.” Over repeated conversations, UCPC clinicians explained how fentanyl “could be making the pain worse” and offered new ways of thinking about pain. This opened space for her to eventually say, “You know what? I want to try this.”

External circumstances also influenced decisions. In one case, a participant’s access to her opioid medication was abruptly removed due to reasons outside of UCPC, prompting her family physician to consult UCPC and initiate buprenorphine/naloxone. Another participant highlighted cost as a factor, reporting that buprenorphine/naloxone was less expensive than hydromorphone.

### Theme 2: Delays to starting buprenorphine/naloxone and system-level barriers

Three participants reported substantial delays between deciding to start buprenorphine/naloxone and actually receiving it. These delays were attributed to prescriber training requirements, screening processes, and perceived bureaucracy. Specifically, the College of Physician and Surgeons of Saskatchewan requires physicians to complete specific training before prescribing buprenorphine/naloxone.

For some, this took months. One participant described waiting the entire summer before her family physician completed training:

And we spent the whole summer trying … waiting for my doctor to take the training so she could prescribe it for me. ’Cause she didn’t have what she needed to do that. So it was September before we go to which was hard because in June I was fired up and ready to go, and as it went long and longer waiting, it was harder and harder and I started to doubt myself.

Another participant reported a delay of nearly a year and perceived this as linked to repeated efforts to ensure she truly wanted to switch: “They keep on asking you questions to make sure you want to try it, make sure you want to try it to keep on, just asking questions. And I don’t know, it took me almost a year.”

Access barriers also shaped experiences. One participant had difficulty obtaining buprenorphine/naloxone consistently due to pharmacy stocking issues and found that fentanyl was easier to access. Another described restrictive insurance policies that limited dispensing to a 7-day supply:

It’s my own insurance company that caused the most issues. … I’m only allowed to get seven days’ worth of pills at a time. … [My pharmacist] said if someone comes in with an addiction, they’ll let her bill a month at a time, but for pain management … they’re not. … So I haven’t had a great experience with them.

Despite generally positive interactions with clinicians, some participants felt that information about buprenorphine/naloxone’s likely effects was incomplete or overly optimistic. Two participants described feeling misled about its expected impact on pain.

One felt that there was false hope offered in terms of its effectiveness:

I kind of feel like I was kind of blindsided a little bit too. I think they like to get people off of the opiates and that there, and I think they kind of proffer this false hope and it gets people off because they’re willing to try something different and they want to get off. But then in my case, I found out that it was one of the biggest grifts going out there because it didn’t do anything for my pain. It just moved me over from one thing that didn’t work to another thing that didn’t work.

Another felt the switch was essentially a lateral move: “I mean, all you’re doing is replacing one opioid with another in the end. So I don’t know if there’s anything that I could really say good about it.”

Participants also noted uncertainty about the long-term plan: how long they would remain on buprenorphine/naloxone, what tapering might entail, and what options existed if the medication became less effective over time. For some, these unanswered questions contributed to ambivalence about the transition and, in one case, to discontinuation.

These delays, barriers, and information gaps were discouraging and, at times, undermined motivation to transition or continue treatment. By contrast, other participants reported relatively rapid transitions, beginning buprenorphine/naloxone within 1 to 2 months of initial discussions.

### Theme 3: Supports and facilitators to switching

Some participants were initially hesitant to change medications, particularly if they perceived their opioid as still helpful or feared withdrawal and side effects. Supportive relationships with knowledgeable clinicians and family members were key facilitators.

Participants described UCPC clinicians who provided clear information, reassurance, and a sense of safety. One participant referred to the promise that she could return to her previous regimen if buprenorphine/naloxone did not work as a “security blanket.” Physician follow-up, such as regular check-ins, also helped sustain confidence during the transition.

Family support played a meaningful role for several participants. Spouses and adult children were described as partners in decision making, helping participants weigh risks and benefits, managing day-to-day challenges, and staying committed to the process. For others, personal values and experiences strongly motivated change. One participant identified as a recovering addict and did not want to stay on an opioid long-term. Another did not want to have opioids in her home because her son, who lived with her, was recovering from a substance use disorder.

Many participants expressed appreciation for these conversations and supports, though several wished they had learned about buprenorphine/naloxone as an option much earlier in their pain trajectories.

### Theme 4: Transition experiences and challenges

Most participants (*n* = 5) tapered off their previous medication while slowly introducing buprenorphine/naloxone. One participant could not recall details of the protocol, and another had a forced, abrupt cessation of their opioid prior to buprenorphine/naloxone starting.

Transition experiences varied widely. Three participants described relatively smooth transitions with minimal withdrawal symptoms, although one described a particularly difficult day early in the process:

Second or third day, I went through a tough day pain-wise, and I can remember thinking that I didn’t think this was going to last. I didn’t think I was going to go through with this, that I was going to stop. But by the next day I was feeling better and virtually went through the rest of it.

Even among those with positive experiences, participants noted the need for patience and cognitive reframing regarding pain expectations and the role of medications. One participant described a clear “revelation” about day-to-day improvements: “Let’s say the ninth or tenth day, I had a revelation mode where I realized that something really was changing and that I really was feeling better in ways I just didn’t really recognize to start with.”

Others adjusted expectations downward. One participant was initially excited and gave the medication 6 weeks to prove itself, feeling that it worked well at first but later required dose increases. One participant praised the logistical organization of the process but felt the expected benefits were never clearly articulated and ultimately never materialized:

I mean, they were very nice people and very thorough about the process and whatever. We didn’t generally—they didn’t really talk much about what it was going to do for me, though. That was interesting. I mean, it was all more—the conversations were more logistical and about the process and stuff. They didn’t really say, “Okay, when you switch over from this to this, this is what we expect to happen.” That never happened. And I guess rightly so, because, I mean, nothing did happen.

Another participant experienced withdrawal symptoms during the transition but felt strongly supported by the UCPC team, including regular check-ins and access to a social worker and a physical therapist: “I had great support from the team, so that was very helpful and made a big difference.”

One participant had significant illness during their transition (e.g., vomiting), but once she figured out the timing and dosing, about 1 month in, she felt a lot better. Another initially felt well but developed persistent side effects and minimal pain relief within a month and ultimately discontinued buprenorphine/naloxone.

Two participants described mood-related changes during the transition, including irritability, low mood, and anxiety that affected relationships: “Everybody noticed my personality and stuff changed. I was very short with people and just nervous, I guess, a lot of times with it.” Another participant experienced increased anxiety around the time of the switch, which they felt may have reflected the combined impact of multiple medications and withdrawal from their previous opioid.

### Theme 5: Side effects

In addition to the transition challenges described above, participants reported a range of side effects. The most common was drowsiness (*n* = 3), although one participant noted difficulty sleeping after a dose reduction:

When I was coming down on my dosage from 4 mg at night to 2 mg at night. Since I was less drowsy on 2 mg, I had a really hard time falling asleep at first, probably for a week or two. I had to contact my psychiatrist and get one of my other sleeping medications bumped up in order to sleep with that lowered dose of Suboxone. After a couple weeks, things leveled out again, and I was able to fall asleep and stay asleep quite soundly.

One participant experienced constipation and vivid dreams, and another reported mood changes that ultimately led them to discontinue the medication. For one participant, depressive symptoms and emotional “anguish” were the primary reasons for discontinuing buprenorphine/naloxone, particularly in the absence of meaningful pain relief: “If it helps with the pain, fine. But like I said, the mental anguish that it put you through, to me is not worth it. And that’s their strong selling point is it’s going to help you with the pain, but there is no mention of what kind of negative effects it could be on it.”

### Theme 6: Pain, quality of life, functioning, and stigma

Participants’ appraisals of buprenorphine/naloxone were shaped by its impact on pain, mood, daily functioning, and overall well-being. Experiences ranged from transformative improvement to minimal benefit or disappointment.

Psychological and physical responses varied. Four participants reported no noticeable changes, though one of these individuals was discouraged by the lack of pain relief. One participant experienced nausea and vomiting that later subsided. One participant attributed new mood changes and headaches to buprenorphine/naloxone.

Several participants described meaningful positive changes. Improvements in mood, appetite, and daily functioning were often linked to enhanced pain control. One participant described a period of profound relief:

I actually went through a period of time there where I would tell my family, I’m not high on this drug because I was so giddy and just to show my husband, I would then dance around the living room and do silly things like that and just truly felt like I had a life back that I hadn’t seen for many, many, many years. Never, ever thought I could have that back.

This analgesic effect lasted approximately 7 months and the participant noted that she no longer has “the same absolute joy and freedom that I had for that period,” with the pain returning but not at the intensity experienced before transitioning. She stated, “I have this pain and it is bad but it’s just not in the same ballpark.” Similarly, another participant reported excellent outcomes after transition and noted “it’s only the last about 2 months that I feel that I need to increase.” This participant had been on the medication since 2022, indicating effective pain management for at least 2 years.

For others, benefits extended beyond analgesia: one participant found that the medication reduced anxiety, and another recovering from OUD felt it helped manage cravings. The latter expressed uncertainty about whether discontinuation might retrigger cravings, reflecting mixed guidance she had received from her providers.

Four participants reported broader improvements in quality of life, such as increased ability to perform daily tasks, greater mobility, and renewed engagement in social activities. One participant noted that although anxiety had emerged during the transition, she believed the medication was helping her manage it, even as she struggled with the trade-off between pain and anxiety. Two participants, however, reported no improvement or worsened well-being due to mood-related side effects.

Female participants who experienced pain relief also described notable functional gains, including doing household tasks, exercising, and comfortably leaving home for errands or social activities. For example, one participant stated, “I can talk to people now and I can go out to different activities, and it’s kind of freed me to even—I feel safe enough even going for a drive by myself to meet up with my girlfriend or something.” Another noted, “Like I said, I can be more active and more able to do things with my daughter. And my stamina … being able to stand for longer periods of time without having to sit.” One participant credited physiotherapy for some improvements but emphasized that buprenorphine/naloxone’s lack of sedative effects enabled her to participate fully in rehabilitation.

Perceived analgesic benefit fell into three general patterns. One participant found the medication highly effective. Two participants—both men—reported no meaningful pain relief. The remaining four described partial or situational benefit, with one participant noting that buprenorphine/naloxone “took the edge off” rather than eliminating pain. When asked to rank the effectiveness of buprenorphine/naloxone in managing pain on a scale of 0 to 10, with 10 being the highest, the average rating was 7.4 (mode = 9, median = 8.5).

Stigma was not a major concern for most participants. One felt that stigma had decreased relative to when she was prescribed fentanyl, suggesting that buprenorphine/naloxone may be perceived as more socially acceptable: “Sometimes I wonder if it’s because nobody knows what it is, whereas fentanyl is all over the news.” Another occasionally clarified to others that she was using the medication for pain rather than addiction, but most reported that few people were aware of their treatment.

### Theme 7: Expectations

Participants’ expectations of buprenorphine/naloxone varied and strongly influenced their evaluations. One participant felt the medication “far exceeded” her expectations, citing greater than anticipated pain relief and minimal side effects: “Never expected to have this amount of decreased pain. I found that the transition was good. I found that the side effects were next to nil, so it far exceeded my expectations.”

Others felt expectations were reasonably met once they understood that the goal was improvement rather than cure: “I think it compares pretty well, once I actually knew what it does and how it can help and stuff like that. I kind of had, I don’t know, I would say reasonable expectations going into it, so I think they met the expectations.”

In contrast, two participants who experienced little or no analgesic benefit described feeling underwhelmed or disappointed. One remarked: “If somebody switched me over in the middle of the night when I was asleep and I didn’t know it, I would never know.”

Another had anticipated short-term use and was surprised to find herself unable to stop: “I don’t think that I expected that I would be on it long term … then it became evident that I couldn’t get off of it.”

When asked whether they would recommend buprenorphine/naloxone to someone with chronic pain, four participants said they would (and, in some cases, already had), typically with caveats about individual variability. Two participants who found it ineffective or harmful stated they would not recommend it or would advise others to be “very careful.” One participant felt unable to give a clear answer due to the complexity of her experience and the concomitant medications.

### Theme 8: Suggestion for improvement

Participants also suggested concrete improvements to both the medication formulation and the transition process. Regarding the formulation of buprenorphine/naloxone, four participants stated they wished that the pill had a better taste, was smaller due to its taste, or alternatively larger so that it was easier to split. Several participants also raised broader concerns about information gaps. Though participants generally felt the logistics of switching from LTOT to buprenorphine/naloxone were well managed and supported by their health care team, they emphasized that clear, transparent, and ongoing communication about the medication itself was often lacking.

These communication gaps fell into four main areas. First, some participants felt that buprenorphine/naloxone was presented as simply a replacement opioid, with little explanation of what buprenorphine/naloxone is and how it differed from their prior medication. Second, a few participants reported that side effects and potential long-term challenges were not fully explained in advance. Third, several participants were uncertain about what tapering off the medication might involve, with one reflecting that “I guess they don’t explain to you that it’s really another strong opioid that you’re still going to have to fight at the end to get off of.” Finally, participants questioned what options would be available once the medication’s analgesic effect diminished over time, particularly because some reported a strong initial benefit that later wore off.

In addition to better communication, participants also suggested system-level changes. Four participants emphasized the need for greater awareness of buprenorphine/naloxone as a treatment option, especially among family physicians, both for patients in recovery and to help prevent future opioid-related harms. One participant recommended online information sessions that would allow patients to learn about the medication and ask questions in advance. Others pointed to delays in initiation as a major barrier: one participant suggested that reducing the delay between deciding to start buprenorphine/naloxone and receiving it would improve the overall experience: “When you decide you’re going to do it and when you actually get to do it, not to let that drag on. Because, I came close to just saying no. And that’s more of a head thing than anything else, but dealing with chronic pain has a lot of head stuff in it.” This highlights the mental vulnerability of some patients experiencing chronic pain and the need to be responsive to windows of opportunity for treatment.

## Discussion

To our knowledge, this is the first study in Canada to qualitatively explore the experiences of individuals living with CNCP who transitioned to buprenorphine/naloxone within an interdisciplinary, pharmacist-led model of care. Across a small but information-rich sample, participants described multidimensional changes encompassing pain, identity, relationships, and everyday functioning.

A central finding of this study is the marked variability in how participants experienced buprenorphine/naloxone for CNCP. Though some described meaningful and at times transformative improvements in pain, function, and quality of life, others experienced minimal analgesic benefit or distressing psychological effects despite substantial effort and support during the transition. This tension, between clinical promise and uneven lived experience, represents a key analytic contribution of this study and warrants careful consideration within contemporary chronic pain care.

Participants narrated opioids as more than analgesics: they were markers of illness severity, social stigma, and, at times, of feeling like “not the best person.” This language resonates with literature documenting the moralization of opioid use, where patients internalize stigma linked to both chronic pain and opioid therapy.^25^ One participant contrasted fentanyl with buprenorphine/naloxone as a more socially acceptable option, which aligns with reports that buprenorphine treatment may be perceived as less stigmatized in the context of the opioid crisis.^26^ For one participant, the transition to buprenorphine/naloxone supported identity repair and a sense of “getting life back,” whereas others, particularly those with limited analgesia and mood-related side effects, experienced the change as disappointing or distressing.

Overall, participants’ experiences with transitioning to buprenorphine/naloxone were mixed but generally favorable. Buprenorphine/naloxone was often well tolerated, with mild and manageable side effects for most participants, and was moderately effective for pain, especially among female participants. Several participants described substantial improvements in quality of life, including increased energy and regained ability to engage in activities. Conversely, a subset experienced negative psychological outcomes (e.g., anxiety, irritability, depression) or minimal pain relief, leading one participant to discontinue the medication. These findings are broadly consistent with earlier qualitative work by Edmond et al., who studied buprenorphine/naloxone use for CNCP in veterans and identified themes of satisfaction, patient–provider communication, and the importance of expectations during transitions.^14^ Our findings also echo those of Sedney et al., who reported that many patients viewed switching to buprenorphine/naloxone as a positive change and some described it as “life-saving.”^27^

Participants’ narratives highlight that recovery for people living with CNCP is multifaceted. One participant spoke of a “long recovery.” Though ambiguous, this phrase invites attention to the multiple recoveries at play: recovery from uncontrolled pain, from the functional and psychological consequences of LTOT, and, for some, from problematic opioid use or fear of it. Qualitative work in CNCP suggests that recovery is often conceptualized as regaining agency and valued roles rather than achieving complete symptom eradication.^28^ Our data support this framing: participants who reported benefit frequently emphasized renewed participation in daily life (e.g., household tasks, social engagement) more than numerical changes in pain scores.

Taken together, these findings underscore a critical gray area in CNCP management that is often flattened in clinical and policy discourse. Buprenorphine/naloxone is frequently positioned as a safer or preferable alternative to full-agonist opioids, yet participants’ accounts demonstrate that a more favorable risk profile does not guarantee satisfactory pain relief or overall well-being for all individuals. Rather than functioning as a universal substitute, buprenorphine/naloxone appears to offer meaningful benefit for some while introducing new challenges for others. Recognizing this heterogeneity is essential to avoiding overly simplistic treatment narratives.

Communication quality and expectation setting emerged as central influences on participants’ evaluations of the transition. Patients who described clear, ongoing conversations about goals, likely effects, and uncertainties tended to view the transition more positively. In contrast, two participants felt “misled” or inadequately informed, particularly about realistic analgesic effects, duration of therapy, and side effects, which contributed to dissatisfaction. This underscores the importance of explicit, iterative communication, consistent with prior research highlighting the role of patient–provider dialogue in successful buprenorphine/naloxone initiations.^29,30^

Participants also encountered structural barriers that shaped both timelines and perceptions of therapy. Several described “hoops” (e.g., training requirements, bureaucratic approvals) and pharmacy- or insurer-level barriers (e.g., stock issues, 7-day dispensing limits). They contrasted the relative ease of continuing full-agonist opioids with the hurdles involved with initiating buprenorphine/naloxone. Similar tensions have been documented in opioid agonist therapy research, where safer treatments are often more tightly regulated, potentially disincentivising their use.^31–34^ In our study, delays eroded motivation and, for at least one participant, fostered doubt about whether to proceed. These findings highlight the need to streamline processes, improve formularies and dispensing policies, and support clinicians in offering buprenorphine/naloxone for CNCP.

Although our sample is small, women reported analgesic benefit and functional gains more often than men. This observation may align with evidence that women can respond as well or better to buprenorphine in OUD maintenance and with qualitative work suggesting that women’s pain experiences are shaped by relational coping, help-seeking, and responsiveness to supportive care models.^35–37^ Future research using larger, more diverse samples should investigate whether gendered social roles and expectations interact with the pharmacologic profile of buprenorphine/naloxone and with specific care model features (e.g., pharmacist-led support, virtual care) to influence outcomes.

A key analytic tension is that some participants experienced limited or no analgesia despite growing evidence for buprenorphine’s potential efficacy in CNCP.^5–7,9^ Our findings suggest plausible contextual explanations: misaligned goals (e.g., expectations of complete pain relief rather than improved function and fewer adverse effects), insufficient dose or titration time, co-occurring mood and anxiety symptoms, gaps in communication and follow-up, and structural barriers such as prescribing policies that may interfere with stabilization. Importantly, these mixed experiences unfolded within a broader clinical and policy environment in which transitions to buprenorphine/naloxone are increasingly encouraged, not only on the basis of analgesic potential but also due to concerns about opioid-related harms, regulatory scrutiny, and prescriber risk. One participant in this study was transitioned for a reason that extended beyond pain control (i.e., medication access disruption). In this context, variability in analgesic response becomes especially consequential because patients may feel compelled to accept a treatment framed as safer even when pain relief is uncertain.

This study has several limitations. First, it was conducted at a single site in Saskatchewan, which may limit the generalizability to other regions or care models. Second, the sample size was small (*n* = 7); thus, the perspectives captured may not reflect the full range of patient experiences. Third, recall bias may have influenced accounts, particularly for participants whose transition occurred more than a year prior to the interview. Fourth, although the interviewer and analyst were independent from the clinical team and lacked medical training, participants may still have felt constrained in their critiques because of their ongoing relationships with UCPC. Finally, the qualitative descriptive approach prioritizes participants’ perspectives and experiential meaning; though this strengthens clinical relevance, it necessarily limits causal inference or claims about comparative effectiveness.

Future research should aim to replicate these findings in larger, more diverse samples and across different clinical models of care. Mixed methods or longitudinal designs could provide deeper insights into buprenorphine/naloxone transition experiences over time, linking patient narratives to clinical outcomes such as pain severity, function, mood, and medication adherence. Development and evaluation of patient education tools and structured communication strategies, including decision aids and standardized expectation-setting conversations, may help address the informational gaps identified in this study.

Taken together, patient experiences at UCPC suggest that buprenorphine/naloxone can play a meaningful role in the management of CNCP for some individuals, particularly when supported by clear communication, interdisciplinary care, and timely access. Participants who benefited often described improvements in function and quality of life and expressed a willingness to recommend the treatment, whereas others highlighted important limitations. These findings underscore the need for individualized, patient-centered decision making and caution against framing buprenorphine/naloxone as a universal replacement for LTOT. These findings highlight the importance of situating risk reduction within holistic, patient-centered care that remains responsive to the full spectrum of patient experiences and outcomes.

## Appendix. Interview guide

### Interview guide for qualitative study on transitioning to buprenorphine/naloxone for chronic noncancer pain

Adapted with permission from: Edmond SN, Wesolowicz DM, Snow JL, Currie S, Jankelovits A, Chhabra MS. Qualitative analysis of patient perspectives of buprenorphine after transitioning from long-term, full-agonist opioid therapy among veterans with chronic pain. *J Pain*. 2024;^1^:132–141.

**Interview questions**

**Suboxone introduction**

- When did you start taking Suboxone?
- Are you still taking Suboxone? If not, why not?

**Initial opioid use**:
Before switching to Suboxone, what pain medications were you using, and for approximately how long?
Which of those pain medications were stopped when you were switched to Suboxone?
**Reason for switch**:
When you think back, how did you first hear about Suboxone as an option for managing pain?
What prompted the switch to Suboxone?
Who first brought up the idea of trying Suboxone?
What was the reason for wanting to try something different?
How did these first discussions about trying a new medication go? Was there anything particularly good or bad about these conversations?
What do you wish you had known about buprenorphine before trying it?
**Transition experience**:
There are two ways people typically go about starting Suboxone. Either you were asked to completely stop taking any opioid medications, await withdrawal and then start Suboxone or you were asked to take both medications together for a period of time before stopping the opioid. Do you remember which of these methods was used?
How would you describe your experience transitioning to Suboxone?
Did the transition happen when you wanted it to?
Did you experience any physical or psychological discomfort before, during, or after the transition? If yes, could you describe?
How did you feel the first day after the transition? How about the first week? First month?
Was there anything that helped make the transition easier?
If so, what were those things?
How did you find out about them?

What were the biggest challenges you faced during and after the switch?

Have you faced any stigma or changes in how others perceive you since switching to Suboxone? How have you handled this?

**Pain management**:
How would you say Suboxone works (worked) for you in terms of pain management?
Does it help with pain?
How well would you say Suboxone works for you? If you ranked how well Suboxone works for you with 0 being “it does not work” and 10 being “it works extremely well,” where would you rank it?
Have you noticed any good things about being on Suboxone?
How does Suboxone compare to your expectations of it?
What do you like about Suboxone?
What do you wish was different about Suboxone?
  If someone else with chronic pain asked, would you recommend Suboxone? What would you tell them about it?
**Side effects**:
Have you experienced any side effects since starting Suboxone? How do they compare to the side effects from your previous pain medication(s)?
**Quality of life**:
How has your overall quality of life changed since switching to Suboxone? This can include physical, emotional, mood, and social aspects.
**Functional ability**:
Has your ability to perform daily activities changed since the switch? If so, how?
**Suggestions for improvement**:
Based on your experience, do you have any suggestions for health care providers to improve the process and support for patients transitioning from other opioids to Suboxone?
**Closing question**:
We have covered a lot during this interview. Is there anything that we haven’t covered that you were hoping to share regarding your experiences with Suboxone?

### Demographic questions

Thank you for sharing everything you have. In closing, we have a couple of quick questions to ask you to help us describe the participants of this study. As a reminder, all of the information shared from this study will be done in a deidentified manner. You can decline to answer any of these questions.

- What is your age?
- Do you live in an urban or rural area?

### Closing statement

Thank you for sharing your experiences and insights with us. Your feedback is crucial in helping us understand the long-term outcomes of switching to Suboxone and improving chronic pain management practices. If you have any additional thoughts or questions after this interview, please feel free to contact us. Once we have transcribed the interviews, we will provide you with a copy of the transcript and will await your approval before proceeding with the data analysis. Your participation is greatly appreciated.
